# Exploring the design of a sport for employability program: A case study

**DOI:** 10.3389/fspor.2022.942479

**Published:** 2022-10-25

**Authors:** Tessa Commers, Marc Theeboom, Fred Coalter

**Affiliations:** Sport and Society Research Unit, Department Movement and Sport Sciences, Vrije Universiteit Brussel, Brussels, Belgium

**Keywords:** sport for employability, NEETs, theory of change, Flanders (Belgium), sport for development

## Abstract

Rates of young people who are neither in employment, education, or training (NEET) are fairly high in the European Union. Correspondingly, there has been a growing tendency to regard sport as a suitable tool to develop soft skills and raise NEETs' level of employability. However, if and how such sport for employability (SfE) programs are able to realize these major claims has been called into question. The purpose of the present study was, therefore, to explore how an actual SfE initiative constructs and delivers its program. In addition, the article assessed whether the investigated program operates in line with researchers' recent calls for theory-based approaches. Guided by a case study approach set up within an initiative located in Flanders, data were gathered through 12 semi-structured interviews with 8 program providers. Results, analyzed using thematic analysis, indicated that the program was characterized by an absence of well-defined desired outcomes, imprecision as to how the program should contribute to these outcomes, and consequently minimal attention to the follow-up of participants' progress. As such, these findings and the accompanying challenges point to the absence of a theory-based approach. Several possible sources for the lack of a systematic approach are discussed.

## Introduction

According to Eurostat (1), over 13 million or 17.6% of European youth aged 20 to 34 years were neither in employment nor in education or training (NEET) in 2020. Although the number of these young NEETs has slightly decreased in recent years, this situation of potential disengagement among a large group of youth in Europe remains a societal challenge. Empirical evidence confirms that NEETs face an increasing risk of poverty, homelessness, poor health, teenage pregnancy, lower life satisfaction, feelings of distrust and crime (2, 3). Moreover, unemployed youth can get caught in a vicious cycle as long periods of inactivity or unemployment can lower their future employment opportunities (4–7).

Different strategies have been proposed to face this societal challenge. One strategy consists of increasing employers' likelihood of hiring young people by providing, for example, wage and training subsidies or tax and national insurance credits (8). Another strategy is directed at (potential) NEETs and includes, for example, investment in good quality and early childhood education and care, intensive support mechanisms from trained advisors, second-chance education opportunities and work-based learning (8–10). All these strategies focused at supporting NEETs are to a considerable extent aimed at fostering employability. Its relevance is also highlighted by European Member States' policy (9). For example, the European Parliament, through the European Social Fund (ESF), attempts to promote social inclusion and combat poverty and discrimination, by focusing on employability: “ESF shall support active inclusion, including with a view to promoting equal opportunities and active participation, and improving employability as an investment priority” (11) (p. 448).

### Soft skills as a central component of employability

Despite the lack of consensus about its conceptualization, some recurring components can be found in most employability definitions. For example, both academic literature [e.g., (12)] and policy related publications [e.g., (13, 14)] refer to soft skills as central components of employability. It has been frequently reported that employers highly value well-developed soft skills, sometimes even more than job-specific technical skills and qualifications (15–19). These soft skills (e.g., interpersonal skills, timekeeping and problem-solving skills) will consequently help NEETs to develop more trainable specialist or technical skills on the job (14, 20). Moreover, soft skills can help them to cope with daily stressors and negative experiences (such as for instance poverty, bad housing conditions and substance abuse). Such stressors may lead to feelings of incompetence and low motivation and ambitions with regard to education, employment and future ambitions (21, 22). As such, the acquisition of soft skills, with a specific focus on positive psychological capital (hope, optimism, perceived self-efficacy and resilience), may help NEETs in dealing with their vulnerable situation (18, 23).

### Sport as a proposed solution

Sport is proposed as a suitable tool to develop the aforementioned soft skills. It is argued that sport can contribute to the development of such skills as communication, teamwork, self-motivation, perceived self-efficacy, self-discipline, problem-solving and perseverance (24–32). Accordingly, an increasing number of initiatives aim to use sport as a tool in combating high levels of youth unemployment (33). This trend is also apparent within and prompted by transnational, national and local policy, which serves to legitimate sports' supposed relevance to increase employability (34). For example, the European Parliament states that: “Social inclusion, social function and accessibility of sport underlines the value of transversal skills acquired through sports as part of non-formal and informal learning, and further stresses the link between sports, employability, education and training” (35) (p. 11).

Such initiatives are captured under the rubric of 'sport for employability (SfE) practices' and can be regarded as a specific subset of sport for development (SfD). However, given the limited number of studies concerned with increasing employability through sport (34, 36), we will first draw on literature from the broader field of SfD.

### Sport for development and its black box

Sport is claimed to contribute to numerous social, educational and health goals (29). It is believed to possess an inherent quality of goodness and purity (37) and is used in a wide variety of intervention programs that focus on personal development (38). However, this potential of sport is not unconditional. Among others, Coakley (37) critiques the widespread idealized and unquestioned beliefs about sports' developmental role. These beliefs are often based on the idea that merely participating in sport will provide a simple and cost-effective tool for improving the life quality of individuals and solving a number of societal problems. Various authors have indicated that such assertions neglect the lack of empirical evidence (39–43) and overlook potential deleterious consequences of sport participation, such as emotional abuse (44), interpersonal violence (45) and alcohol (46) or doping (47) abuse. Rather, Coakley (37) highlights the specific factors and conditions that may influence potential outcomes. For example, he refers to “settings where young people are physically safe, personally valued, morally and economically supported, personally and politically empowered, and hopeful about the future” (p. 310). This correlates to the fact that sports-based initiatives should be about much more than sport if they aim to achieve positive developmental outcomes. These developmental outcomes will be dependent on the context that is created within and beyond the sport sessions. Hartmann (48) even states that “The success of any sport-based social interventionist program is largely determined by the strength of its nonsport components” (p. 134). As such, Coalter (49) calls for acknowledging sport as a necessary but not sufficient condition to reach any developmental potential.

Based on the prominence of the social role of sport, Coalter (39, 49) distinguishes between *Sport, Plus Sport or Sport Plus*. Sport can be organized just for the sake of sport. But when sport functions as a ‘fly paper’ to engage targeted youth within programs of education and training, it is labeled as *Plus Sport*. A fairly different approach is reflected in *Sport Plus* practices whereby sport is not only deployed to attract targeted youth, but sporting activities are tailored and supplemented by non-sporting activities (e.g., workshops) to achieve certain developmental goals. Both *Plus Sport* and *Sport Plus* approaches explicitly use the social values of sport.

Despite sports' social or developmental potential depending on its intentionally created development opportunities [e.g., (47)], SfD programs remain black boxes without a clear understanding of critical success factors required to achieve specific and predefined outcomes (39). Most social programs are designed based on “experience, practice knowledge, and intuition” (50) (p. 503) and the involved practitioners “go about their work without articulating the conceptual foundations of what they do” (p. 503). In accordance, SfD research is characterized by a general lack of robust evaluation (40, 49, 51, 52). Recently, a number of reviews aimed to summarize the growing amount of SfD studies [e.g., (35, 53, 54)]. However, these reviews do not consider the quality of evidence of the original studies. A critical evaluation of SfD studies by Whitley et al. (51) revealed that (a) most studies do not contain enough methodological details to perform a critical assessment and (b) the quality of methods is described as rather weak. Moreover, research and practice on this topic focuses almost exclusively on intervention outcomes and pays limited attention to the critical factors that produce these outcomes (21, 37, 51, 54, 55). Not only does this preclude rigorous testing (and improvement) of programs, a limited understanding of program processes also inhibits intentionally working toward aimed outcomes and impacts (51).

### Need for theory-based approaches

Therefore, several researchers pointed to the importance of providing insight into the process of SfD programs by using a “program theory” or a “theory of change” [e.g., (50, 51)]. According to Weiss (56), all policies or initiatives have an implicit idea or theory that precedes the implementation of their initiative. “Theory” does refer to these implicit or explicit ideas about how and why a program will work or how doing “A” will result into “B” as the desired outcome. Consequently, it is important to note that “theory” does not refer to any specific scientific (psychological) theory. However, particular scientific theories will always be involved as they do underpin and explain possible expected changes within the chain of reasoning.

Such a theory-based approach “seeks to identify the components, mechanisms, relationships and sequences of cause and effect that are presumed to lead to desired impacts and outcomes” (49) (p. 53). In other words, it seeks to enter the black box of SfD programs and articulate how programs aim to achieve specific outcomes. This process of constructing a theory of change therefore presupposes getting to know the desired impacts or outcomes and explain in as much detail as possible how you can achieve them (57) or making explicit the often unspoken underlying assumptions that undergird any program and its constituent activities (50).

A theory of change ideally starts with the aimed impact of the program and contains short-term outcomes and outputs, the various activities of the program, and the resources or organizational inputs required to implement these activities (58). Characteristic of a theory of change approach as suggested by Weiss (56) is that it is situated at the level of the program and is articulated, owned, and approved by the program's stakeholders. Moreover, the theory provided should be plausible (i.e., correspond to what is already known), doable (i.e., realistic in terms of available resources), and testable (i.e., sufficiently specific and complete which means an evaluator can verify the information) (58). While Weiss makes a distinction between ‘implementation theory’ and ‘program theory’, theory of change is mainly concerned with making explicit implementation theory (57). This involves the daily implementation or operation of the program. It articulates which program activities are needed to achieve the program's aimed objectives. Program theory, on the other hand, is more concerned with mechanisms that are triggered by the program or participants' responses to the program activities. In essence, theory of change is concerned with the general outcomes of the program, how these outcomes relate to the different components of the program and how they mutually interact. It therefore, brings simplicity and direction to a complex program and is often depicted in a visual manner (59).

Several benefits are linked to this theory-based approach. First, it offers the possibility to intentionally and commonly stimulate these outcomes or impacts by focusing on the conditions and mechanisms that are responsible for triggering them (60). Second, it may help program designers to articulate “theoretically coherent, realistic and precise impacts related to program processes and participants” (52) (p. 21). In addition, it helps program designers by offering more insight into why (or why not) any changes occur (52) rather than merely focusing on the simple question of whether an intervention “works” (50). Third, having an insight into the program assures program providers can evaluate the initiative based on processes and outcomes that the initiative intentionally tries to promote instead of measuring arbitrary outcomes that might be affected by factors outside the program (49). It thereby aims to overcome issues related to experimental testing as this does not offer any information on the context in which the program was delivered. Finally, the explanation of how inputs can be connected to certain impacts sounds like a story of the program and can therefore be very much appreciated by some policy makers (50). As demonstrated by these possible benefits, a theory of change approach contains the ingredients to more effectively and efficiently stimulate positive changes. Despite these benefits, the use of theory-based approaches is, contrary to other domains such as health promotion (61), not common within SfD (51).

### Developing SfE research

According to Coalter et al. (62), sport constitutes only one component within SfE programs. Sport is combined with other activities that assist in meeting the goal of employability. Activities include workshops (focused on job coaching or broader social themes) or complementary activities such as getting in contact with employers and labor-related organizations. Within this specific and relatively new subset of SfE, few studies have recently empirically examined how programs can be effectively implemented. Walker (33) studied the contribution of an employability program by drawing upon Basic Psychological Needs Theory. However, the study does not provide any insight into the rationale of the program providers. In line with the need for theory-based approaches within SfD, two studies have incorporated this in their study design. Warner et al. (63) conducted a feasibility study of three evidence-informed and collaboratively developed and delivered SfE programs. They concluded that collaborative development of SfE programs might be considered as a promising strategy to increase participants' employability. Within the most recent study, Coalter et al. (62) provided a better understanding of program processes by developing a generalizable program theory for SfE programs for NEETs. Their model is based on perspectives of both program providers and former participants of 10 selected organizations.

Although the current literature provides valuable information based on an analysis across different programs, in-depth insight into the workings of a particular existing SfE program remains absent. Thus, the purpose of the present study is to take a closer look at a SfE program and explore how it structures and organizes its program in an applied setting. Such a detailed case study analysis (64) might provide in-depth insight into how current and existing practices operate, including the potential challenges they might encounter. Therefore, the first research question is: how does a SfE program construct and deliver its program? More specifically, based on common components of SfE programs, we are interested in: (a) How does a SfE program define the desired outcomes of the program? (b) How does a SfE program work toward achieving the desired program outcomes? (c) How does a SfE program monitor and evaluate the level of progress of the participants? Given the illustrated absence of insight into program processes within SfD, the secondary research question is: to which extent does the investigated SfE program operate in line with theory-based approaches?

### Context

This study adopted a case study approach to address the research questions. Data were collected within a municipal sports-based employability program in Flanders, the Northern Dutch-speaking part of Belgium. Data of this municipality (from 2019) show that only 65.4% of the local population between 18 and 64 years old is employed (65). This number is below the Flemish average of 75.2% and the lowest of all 13 main cities in Flanders (66). More specifically, 19.9% of the 15- to 24-year-olds living in the area are registered as unemployed jobseekers, which is distinctly higher than the Flemish average of 14.8%. It should be noted that this is an underestimation as this number only refers to registered jobseekers. Other indicators such as population of foreign origin (58.5%), early school leavers (17.5%), and children who live in a socially disadvantaged situation (23.2%) also demonstrate that, compared to the Flemish average, there is a considerable higher presence of people with a vulnerable position in the labor market (65).

In 2017, the municipal employment service and a social sport initiative (which can be considered as the main program organizers) joined forces with several local partners and initiated two SfE programs targeting NEETs (one male, one female). While the project description of the male program specifically aimed at young adult males (between 18 and 30 years old) unknown by the Flemish employment service and therefore missing opportunities for support to find work, the female program targeted women (between 18 and 50 years old) lacking the skills and resilience conducive to their integration into the labor market. This also included their low Dutch language level. The program was organized three times a week and lasted 10 (female) or 20 (male) weeks. The programs had different lengths as the specific organizers of the women's program thought 20 weeks was too long to keep participants motivated. The programs included various workshops and activities related to (non-sports-based) experiential learning (cooperation and outdoor activities complemented by reflection), sport, job coaching and social orientation and additional job coaching support if needed. The sessions experiential learning accounted for half a day per week within both programs and consisted of, among others, climbing, cooperation and problem-solving games (e.g., games whereby trust and clear communication where crucial when guiding a blindfolded participant or by means of a walkie talkie). Sport sessions were more prominent in the male program as it covered a full day per week compared to half a day within the female program. These sessions included both team and individual sports (e.g., football, boxing, swimming, body shaping and netball). The final combined component of job coaching and social orientation was in the female program organized during one and a half day per week and one day within the male program. In general, the content of this final component covered informal training on social skills, work attitudes, labor market expectations, personal strengths and gaining insight into labor-related organizations. Example activities include a game about one's personal strengths, a quiz on labor market expectations, interview training, and a visit to the Flemish employment agency. The activities were distributed equally over the duration of the program. Evidently, the specific content of the activities became more labor market oriented as the program progressed (e.g., practicing job interview skills was addressed at the end of the program). The aim of both programs was to enter the labor market in a sustainable manner. Twelve female participants participated in the program and were always present over the course of the program. Within the male program, an average of 15 people attended the program. During the course of the program, the number of male participants fluctuated as some participants dropped out, while others entered the program at a later stage.

## Method

This study used a case study approach as it facilitates an in-depth exploration of phenomena (67) and can be considered as a suitable strategy to study research questions focusing on how and why (68). The selected case agreed to let the first researcher immerse herself in the SfE program. This is in line with Stake (69) who argued that choosing an accessible case where you can conveniently spend time offers the best learning opportunities. Moreover, the initiative could be considered an appropriate case as it was one of the very few examples in Flanders that organized SfE programs on a regular basis. In addition, the social sport organization, as one of the partners, had a long track-record in using sport to develop social skills.

### Data collection and participants

In total, 12 individual semi-structured interviews were conducted with 8 staff members (4 males, 4 females). Some were interviewed twice as they were related to both the male and female program or to expand on information. Respondents' average age was 36 years (ranging from 26 to 53) and there was some variation with regard to years of experience in the field. The selection covered a variety of staff roles and types of organizations involved in both programs (see Table 1).

**Table 1:** Overview of the profile of interviewees.

**Code**	**Role**	**Years of experience**	**Organization**	Gender^*^	**Age**	Program^*^
P1	Experiential learning coach	2	Social sport organization	M	26	M
P2	Job coach	14	Local employment service	F	41	M
P3	Sport coach	24	Social sport organization	M	53	M
P4	Coordinator partner	3	Women's center	F	27	F
P5	Job coach	15	Local employment service	F	37	F
P6	Sport coach	1	Municipal sports department	F	30	F
P7	Coordinator partner	15	Social sport organization	M	39	M + F
P8	Adult education teacher	10	Center for adult education	M	32	M + F

* Gender: F, Female; M, Male.

The selected data collection method enabled interviewees to report on their own thoughts and feelings and made it possible to obtain a deeper knowledge on their experiences (70). The interviews included open-ended questions related to (1) recruitment and selection of participants; (2) how the program was supposed to work; (3) social climate and mentoring relations within the program; (4) targeted program outcomes and (5) monitoring and evaluation of participants' development. Probing was used to obtain additional information if needed. The interviews had an average duration of 49 min each (SD = 25.53 min) and the research procedure was approved by the ethical committee for Human Sciences of the university.

### Data analysis

All interviews were tape-recorded and transcribed verbatim afterwards. Thematic analysis was used as an accessible method to identify, analyze and report patterns or themes within the data (71). Following the step-by-step guide proposed by Braun et al. (72), the first author started the analysis by reading and re-reading the transcripts several times. This process of familiarizing with the data, which was facilitated by uploading the transcripts to NVivo 12 beforehand, made it possible to code each piece of relevant data. Next, codes or raw data themes were clustered into candidate core themes and were subjected to a process of extensive reviewing and revising. Subsequently, core themes that shared the same concept were grouped into higher order themes. While reviewing these core and higher order themes, the first author went back to the data several times to check for a good fit with both the entire data set and the research questions. Finally, higher order themes were clustered into four overarching topics and all themes and topics were inductively defined in a way that captured their core content. When distinguishing between the different approaches of thematic analysis, the adopted procedure and underlying philosophy can be considered to be in line with “reflexive thematic analysis” (73). This means analysis was a situated, interpretable, and reflexive process whereby the subjectivity of the action should be considered as inevitable and as a benefit instead of a constraint. It also implies that coding of the data was done in an open and organic manner. Consequently, the analysis did not pursue early theme development, did not make use of any coding framework, and did not strive for inter-rater reliability.

Several strategies were used to facilitate the trustworthiness of the study (74). Prolonged engagement during an 8-month period by strategies of participant observations and document analysis of the program's policy documents served two objectives. First, participants' familiarity with the lead researcher established rapport with the participants and created a relationship based on trust and respect. Second, it supported a deeper understanding of the data (75). In addition, trustworthiness within this qualitative study was ensured *via* a critical friend (second author) who offered a theoretical soundingboard by eliciting critical dialogue and encouraging reflexivity on the lead researchers' interpretations (76).

## Results

The thematic analysis of the data provided insight into how the SfE initiative constructs and delivers its program and to which extent it operates in line with theory-based approaches. This resulted into four topics, each divided into several themes. Three of these topics (desired outcomes of the program, program approach, monitoring and evaluating the level of progress of the participants) were in congruence with the research questions. Furthermore, a fourth topic, relating to the integration of stakeholders, was added during data analysis as it provided important information to understand how the program operates. The relationship between the different topics, higher order themes, core themes, and raw data themes is illustrated in Table 2 and will be discussed in more detail below.

**Table 2:** Overview of the SfE program construction and delivery.

**Raw data themes**	**Core themes**	**Higher order themes**	**Topics**
Perseverance			
Oral communication	Existence of list of outcomes		
Motivation			
No consultation program providers			
Limited impact on program design	Lack of well thought outcomes		Desired outcomes of the program
Primarily designed for project funding			
Adopted from other project			
Policy wants quantitative data	Political pressure to present		
Rejection of presenting nuanced results	positive outcomes		
Own network	Extensive recruitment through		
Local organizations targeting NEETs	various channel		
Coming from the municipality			
Between 18 and 30 years old	General idea intended target group	Recruitment and selection	
Having a distance from the labor market			
Selection was not conscious process			
Procedure was not clearly defined	Lack of selection guidelines		
Failing attract intended target group			
Raise self-awareness through experience and reflection			
Process of recognizing, acknowledging, and exploring	(non-sports-based) Experiential learning		
The delivery of sessions was hampered by irregular attendance			Program approach
Expected to trigger several positive outcomes		Main program activities	
Sport was used differently within both programs	Sport		
Sport was insufficiently used as a learning tool			
Collective sessions included three parts			
Ambiguities regarding individual guidance	Job coaching and social orientation		
Program shuffling regarding specific activity and its content			
Content driven by emerging needs or topics	No adherence predetermined schedule	Structure and integration of the program	
Separate program activities and approaches	Minimal integration of the different program activities		
Lack of alignment			
Safe social climate			
Establishing trusting relationships with participants	Social relationships as success of the program		
Unconditional support	Divergence of mentoring styles	Mentoring and social	
Critical support focused on empowering		climate	
More credibility coaches equal to them	Distinctive relationship based on perceived similarity		
Difficulties conducting a formal diagnosis of participants' personal situation	Missing a systematic approach M&E participants' progress		Monitoring and evaluating the level of progress of the participants
Difficulties to include goal setting			
Lack of systematic monitoring system			
Perception relying on intuition			
Unclear when participants are expected to reach this goal	Difficulties determining employment status		
Need for cooperation respecting rationale program	Minimal integration referral agencies		Integration of external stakeholders
Solely bringing into contact with employers	Minimal integration potential employers		
Focus on participants' own ability			

### The desired outcomes of the program

The organizers listed 18 intended program outcomes. The list covered psychosocial outcomes including, among other things, perseverance, oral communication, motivation, stress management, attendance, and initiative. However, a follow-up conversation with one of the providers revealed that the list was not based on an actual consultation with the other program providers and was not consistently put into practice (2018 Mar 5 telephone conversation from P7 to me; unreferenced). It was developed intuitively and primarily designed for the project to be submitted for funding.

Furthermore, providers reported that they experienced political pressure to modify the program results in a positive way. The coordinator of one of the partners argued policy wanted to present simple evidence, including figures of those who access the labor market, without taking into account participants who may not fit into the project: “that's a bit of a trap with this kind of interventions, when you look at policy, they obviously want quantitative data” (P7).

### Program approach

#### Recruitment and selection

The program organizers had a general idea with regard to the intended target group, but recruitment and selection was not a conscious process and the selection procedure was not clearly defined. Extensive recruitment was done by drawing on its own network of potential participants and through contacting and promoting the program among specific local organizations who are likely to target NEETs and vulnerable groups (e.g., youth work organizations, public social service, local job center). A number of general selection criteria were used for the inclusion of participants in the program. Reference was made to “coming from the municipality”, “between 18 and 30 years old” and “having a distance from the labor market”.

However, interviewees stated that selecting participants was not a conscious process: “That is not a deliberate decision. (…) I find it very difficult to say who I do and do not select and why” (P5). Accordingly, the selection procedure was not clearly defined. Although a selection day (including intake interviews and a cooperation assignment) was organized, participants could enter the program at any time during the whole program and without being formally selected on the selection day: “In the beginning, it was a mess: who will come and who will not and based on what do you participate in the program?” (P1).

The fact that clear selection guidelines were lacking also resulted in some cases in failing to attract people with a distance from the labor market: “Our local authority says 6 out of 8 former participants are currently working in a sustainable manner. That's basically correct, but they shouldn't have been in this project as they would have found employment anyway” (P7).

#### Main program activities

Both programs consisted of three major activities: (non-sports-based) experiential learning, sport, and job coaching and social orientation. Respondents indicated the aim of the experiential learning sessions was to raise participant's level of self-awareness. Experiences such as cooperation and outdoor activities were complemented by a reflection component where participants were encouraged to reflect on and discuss their own attitudes and behavior. According to the experiential learning coach, an underlying process of recognizing, acknowledging, and exploring was intended to approach things differently in the future:

For instance, x is a participant who encounters many difficulties at work and is only able to complete short contracts. Here we see someone that complains a lot and is always making remarks. That is mentioned and discussed. During the one-week training camp, the other participants also mentioned: “grumpy bear is at it again”. If a person acknowledges something, he or she can see what the effects are and then you have the choice to do something with it. (P1)

The coaches explained that the experiential learning sessions were characterized by a specific structure focusing first on “creating a safe learning environment and getting to know each other” (P1), while gradually shifting the emphasis to incorporating reflection and developing soft skills. However, a major barrier within this process consisted of irregular attendance of some participants. As one of the coaches stated: “This made it difficult to use the group as a tool and created a constantly fluctuating group dynamic” (P1).

Another main component of the program related to the sport sessions, which in the male program intended to be used as, among other things, an attraction pole or “flypaper”. Furthermore, respondents highlighted that involvement in these sessions was expected to trigger several positive outcomes, including feeling better, enhanced social skills (e.g., listening to others, daring to speak in front of a group), enhanced perseverance and improved group cohesion. While referring to broadly the same suggested positive outcomes, the interviews revealed that sport was used differently within both programs. The male sport sessions included, among others, football, running, boxing, climbing, swimming and fitness. The program providers' accounts reflected that sport was intended to be used as a learning tool but did not reach its full potential as many learning opportunities were left unused. It was believed that a lack of expertise was primarily responsible for this: “Here you can work more deeply and on an individual base compared to a regular sport class. But that's still a learning process for me. I feel I need more background and frameworks to apply this even more” (P1). Where reflections were included, they rather took the form of “how did it go?”. The only notable exception related to a session whereby participants had to teach a boxing lesson to an external group. In this case, participants were instructed to specifically focus on job interview skills such as “speaking loud and clearly” and “a proper appearance”. Afterwards, they concluded with a reflection including feedback from the people who received the boxing lesson, the other participants of the program and themselves.

Female sport sessions included, among other things, body shaping, fitball, boxing, badminton, and netball. Coaches believed that participants would be more interested in feminine sports and therefore “consciously opted for more feminine sports such as Zumba and fitball” (P7). Although reference was made to a number of expected positive outcomes, a number of interviewees explicitly stated that the role of sport was rather ill-considered:

We did insufficiently deploy sport as a means to develop skills. Things happened and were discussed afterwards but this was all on an *ad hoc* basis. It wasn't originally the idea to use sport to enhance employability. I think we had too little thought about it beforehand. (P7)

Furthermore, it is interesting to note that there was a lack of common understanding regarding the role of sport within the program. When asked why sport was part of the program, the sport coach replied: “You have to ask them [the municipal employment service and the social sport initiative] why sport is part of the program, the main program organizers didn't tell me” (P6). Program providers, for example, stated that reflection or a link with a work situation was not part of their sport activities: “There is little explanation regarding sport. The morning already requires reflection during the sessions experiential learning, so we'll just let the afternoon be relaxing” (P5).

The final major component of the program involved job coaching and social orientation and consisted of both group sessions and individual guidance by a job coach. Collective sessions included three parts: (a) understanding participants' interests and aspirations, (b) support with CV writing, interview training and job searching, and (c) an introduction to various sectors and jobs including visits to potential employers and labor-related organizations (e.g., Flemish employment agency, trade unions, training institutions). Subsequently, intensively working toward work, education or any other suitable goal tailored to the participant (e.g., volunteering) was the key aim of the individual support provided by the job coach. However, this individual phase was not quite specified in advance and therefore “not sufficiently integrated within the intervention” (P2). This ambiguity was also reflected in both the format, frequency, and duration of the individual job coaching support. To illustrate, the job coach of the female program described this phase as: “There is no fixed period on how long I guide them” or “For some participants I go on home visits and for others I don't … some need more guidance than others” (P5).

#### Structure and integration of the program

Despite the existence of three major activities, there was no adherence to a predetermined schedule in terms of the particular activity and its content. This was illustrated by some of the coaches: “There was written ‘sport’ within the planning, but when each part was delivered was often determined on an *ad hoc* basis. We did a lot of shuffling within the planning” (P7). Instead, providers stated that the content of the program was mainly driven by emerging individual and group related needs or topics, rather than deliberately integrating explicit learning opportunities based on predetermined soft skills. The following quote illustrates the focus to adapt to the group needs:

It was not fixed within the program when we would address certain issues. Rather, we tried to sense what the group needs and what the group is open to at a certain moment. In the beginning, there was a negative person who made fun of everything and our group was not strong enough to handle that. That could have resulted in a different group dynamic. In order to have that positive group dynamic, it's essential to have a group where you can build upon at that time. (P7)

Respondents also pointed out that there was only minimal integration of the different program activities. Coaches felt that the main activities “remain too much three separate parts that do not form a whole” (P2). This lack of integration, exemplified for instance by some partners' absence from general meetings, resulted in a lack of alignment:

The sessions at the center for adult education [one of the partners] went quicker than we [one of the partners] did. For example, they already put their CV's online and received calls from employers while job search was only on our agenda after 10 weeks. (P7)

Furthermore, there was a difference in approaches between the different program providers of the male program. This difference can be marked by a strong reliance on the method of experiential learning including offering experiences, responsibilities, and reflection on the one hand versus an authority that gives direct instructions without any kind of reflection on the other hand. This difference in approaches became more pronounced over the course of the program and had a hampering effect on the overall coherence of the program. Respondents argued that as not everyone was convinced of the contribution of each component of the program, coaches started to only deliver their own component properly:

Normally, I was supposed to attend more often, also on Mondays. But that was practically unfeasible. Or also because I didn't support those sessions. As for those Mondays, I've taken a step back. The coach of the youth work organization [one of the partners] is doing his thing there and I'll do my thing on Friday. Sitting squatted against a tree, starting to hyperventilate, … I don't see the point. (P1)

#### Mentoring and social climate

Coaches referred to the importance of creating a safe social climate and establishing trusting relationships with participants. In this sense, social relationships were considered to be “the success of the program” (P4). Already since recruitment, there was a strong focus on “feeling comfortable and at home”, “feeling appreciated with both your strengths and weaknesses”, “value one another”, “a caring relationship”, “a sense of belonging” and “always be there for them”. Interested and caring adults and a sense of safety, acceptance and belonging are also part of the “protective factors” as identified by Witt and Crompton (77). These factors are crucial components within programs targeting at-risk youth as they might serve as a buffer against the multiple risks these people encounter within environments such as their homes, schools, or communities. Specific reference was also made to the development of a trusting relationship with the participants. This relationship, as well as the group dynamic that is present within the program, was considered to be even more essential than the content of the program activities. Respondents indicated this relationship originated from sharing an enjoyable experience, which provides bonding and ultimately leads to a relationship based on trust: “When I sing a silly song with them, I'm a friend at that moment and we're doing something ridiculous together. This gives you more bonding and consequently increases the chance to be taken in confidence” (P1). This reflects the presence of “befriending” or the first stage of how a mentor/mentee relationship evolves within a youth mentoring program (78). Participants in our study mentioned that based on these shared experiences, it was possible to discuss certain things, which consequently allowed for insights. More specifically, coaches explained this positive social relationship was a prerequisite to appoint to both positive and negative qualities of participants or could give participants the opportunity to face and discuss their own situation: “One of our women left her husband and that was a trigger to set things in motion. By bonding with them, they dare to express certain issues and you can support them in doing so” (P4). It is believed by the coaches that this environment enabled participants to reconsider their behavior, share personal information and develop throughout the program. This refers to what Pawson calls ‘direction-setting’ or exploring alternatives together with the participants and is only possible after the ‘befriending’ stage or the establishment of mutual respect (see also 40). In this regard, the first stage of bonding or the establishment of mutual respect is a prerequisite for this phase of ‘direction-setting’. Next, the program supported participants to acquire skills and assets that are needed to enter employment or showed elements of Pawson's “coaching” stage of mentoring (78).

In relation to these mentoring styles, a notable divergence became apparent throughout the male program. The interviews indicated that one of the coaches provided unconditional support: “coach x was always there, he did everything for those guys” (P1). As a result, this relationship was marked as “coddling” as “he moved away from the rules” (P7). Most of the coaches, on the other hand, rather underpinned a relationship based on critical support to empower participants. As such, participants should “come to important insights themselves” by “offering experiences” and “promoting responsibility” (P1). According to one of the providers, this imbalance of support between the coaches led to “good cop, bad cop, which causes the program to fail in terms of quality” and “the loss of learning opportunities for the group” (P7).

Another aspect that came to the surface during the interviews was the distinctive relationship that participants developed with coaches that are seen as “equal to them”. According to Bandura (79), perceived similarity gives participants' the confidence that they can master comparable activities and is therefore an important element in strengthening participants' self-beliefs of efficacy. As illustrated by the job coach of the male program, coaches with foreign roots could act as role models and were given more credibility by the participants:

I think the boys look different to me than to coach x or coach y. I think they see them more as equals. You feel the difference. Not that I have a bad relationship but if I wanted to make someone aware “this job is something for you”, if x said that, it had more impact. (P2)

Conversely, coaches who do not seem to meet these requirements were sometimes being told: “It's easy for you, you are not raised in my neighborhood, you don't know what that means” (P7).

### Monitoring and evaluating the level of progress of the participants

Although the initiative was characterized by several promising elements with regard to the monitoring and evaluation (M&E) of participants' progress, it was evident that a systematic approach was missing. This will be explained by successively discussing the different elements (intake procedure, goal setting and M&E of participants' progress) that relate to the stages that participants went through during the program.

Starting with the intake procedure, the male program had no formal diagnosis of participants' personal situation at the start of the program: “It's not like we're evaluating their needs during the selection process” (P7). In contrast, in the female program participants' needs were assessed as part of the intake interview with the job coach:

I have a conversation of an hour to an hour and a half to find out who that person is and what that person needs. “What has caused that you are in the current situation and that you are not getting to where you want to be”. (P5)

The job coach also regularly attended various workshops to get to know the participants, identify their strengths and weaknesses and points that she could work on. Additionally, the program sought to evaluate participants' competences and subsequent development by means of a low-threshold tool. In the absence of a personalized instrument, they looked at existing instruments and were given permission to test a competence measurement tool commissioned by the sport administration of the Flemish government. The instrument was mainly targeted at volunteering in sport but covered the same general target group of people with a certain distance from the labor market. Upon admission, female participants were asked to complete the competence measurement tool representing seven roles or personalities measured by several items encompassing skills and attitudes (e.g., “being able to listen” measured by, for example, the indicator “I let others finish their sentence”). Although results were displayed immediately after finishing the test, results were not discussed in detail with the participants, nor did they provide a basis for setting objectives within the program: “Participants got their results, but we didn't discuss it with them afterwards” (P4).

Regarding goal setting, one of the members of the steering committee expected the female program to work with a personal development plan including goals for each of the participants. For the sole purpose of meeting this requirement, the program providers completed all personal development plans, but this tool was not used within the context of the program as one interviewee indicated “… we didn't do anything with it. […] we never discussed them with the participants” (P4). In contrast, the male program did not have a formally drawn up plan, but objectives were established and subsequently discussed during the individual progress meetings: “Our evaluation, including qualities and areas in need of development, were discussed with the participants. The second time, we included possible changes compared to the previous time as part of the conversation” (P1).

Considering the final element, that is monitoring and evaluating participants' progress, neither program made use of a systematic monitoring system. The initial completion of the competence measurement tool relating to skills and attitudes was not followed by an evaluation on a later occasion within the program: “We didn't do much with the competence measurement tool” (P4). As opposed to the female program, the male intervention did organize some individual progress meetings to discuss participants' development. However, providers of the male program reported that participants still faced difficulties to identify their own strengths: “Naming one's own qualities remained difficult at the end [of the program]” (P1). Coaches stated that they pointed out participants' qualities and development needs, but that they did not subsequently monitor them on a regular basis: “We explicitly mentioned their qualities, but it's not that we looked back at it the following week. Maybe that could have been more concrete. With specific development needs which we might then consider on a weekly basis” (P1).

In addition, the interviews revealed that the decision whether someone is ready to enter the job market is associated with some ambiguity. Reference was made to things such as “participants must first have a strong foundation” and “motivation and attitude are the most important criteria to consider in order to declare participants ready for the job market” (P2). However, the job coach explained that although she did experience a number of differences with the participants concerned, it was hard to substantiate these exact differences. She indicated that it was rather a perception that relied heavily on intuition:

That's pretty much a guess I'm afraid. […] Gradually you feel, but that is some kind of gut feeling, I can send that person to an employer while I did not support that idea in the beginning of the program. Adhere to agreements, punctuality, be realistic, motivation, … […] Those participants that are currently in employment did undergo a few changes. (P2)

Moreover, program providers stated “ambiguity” as to whether participants are expected to reach this goal already before the end of the group program and therefore called for more clarity. Reference was made to former participants who repeatedly dropped out of employment due to recurring issues such as “showing up late”, “an argument with the boss” or “not able to work in a team”. According to one of the providers, the program was partly responsible for this “… as they allow participants to move on when they are not yet ready” (P7).

### Integration of external stakeholders

The program was characterized by only minimal integration with stakeholders (referral agencies and potential employers). With regard to referral agencies which had the possibility to terminate participants' benefits, several respondents reported that the intervention would benefit from a specific cooperation which respects the rationale of the program. This means, providing optimal chances for participants but also supervising certain obligations since the intervention should prepare participants for the labor market.

Similarly, the program did not engage in any specific partnerships with employers. Rather, support provided by the job coach focused solely on the participants and on bringing them into contact with potential employers who originate from the job coach's network. Such a focus on participants' own ability, including building on strengths and removing personal barriers, seemed also reflected within the program: “our program focuses on strengthening the job search: overcoming obstacles and enhancing qualities to make the step toward work easier” (P5). Due to this lack of involvement of (potential) employers within the program, one of the coaches expressed concern as to whether participants were able to transfer any achieved outcomes to the labor market: “it's not clear whether the development of some women has continued within the labor market afterwards, because by then, of course, that safe environment will be gone” (P7).

## Discussion

The aim of the present case study was to gain more insight into how a SfE program defined and worked toward its desired outcomes and how it monitored progress. As a secondary research question, we intended to assess whether the investigated program operated in line with (researchers' recent calls for) theory-based approaches. Our findings revealed that the program providers were driven by high level ambitions such as closing participants' gap to the labor market or education. However, the results mainly illustrated the potential pitfalls or challenges that SfE programs may face. Correspondingly, our findings suggest that the investigated program was designed based on intuition and experience without reflecting on the underlying principles that undergird program activities (56). As such, these challenges point to the absence of a theory of change.

The first observation and challenge related to the absence of well-defined definitions and operationalizations of desired outcomes. Findings suggest that there was limited (shared) reflection on what the program should achieve to sustainably strengthen participants' position in the labor market. As such, the relative lack of focus hindered intentional work toward these outcomes and prevented program providers from evaluating their initiative on the basis of related performance indicators. This finding is in line with research conducted within the broader field of SfD. A number of researchers already concluded that sports' added value is often described in ambiguous, ill-defined, over-inflated and imprecise terms [e.g., (36, 48)]. According to Weiss (50), theory-based evaluation can help programs in formulating short-term and intermediate outcomes who can in turn trigger the ultimate but more long-term outcome. This is also confirmed by Coalter (52) who states that it supports organizations at linking the content of the program with theoretically sound, concrete and realistically expected outcomes. Whereas black box and gray box (discerns components but does not explain outcomes) programs have no understanding of their program or start from program activities, programs characterized by a theory of change start from the desired outcomes of a program and look for proper activities with accompanying responses from participants to attain interim and final outcomes (80).

Second, and in line with the first observation, it was found that the case under study did not use a systematic approach to its program strategy. The content of the program was not well considered with regard to the nature, extent and duration of participants' involvement and contained numerous vague elements. Correspondingly, it was unclear how the program would contribute to the list of intended outcomes. This was exemplified by, for example, unclear selection guidelines to enter the program, a lack of integration of the various program elements and providers, and the unclear role of sport. According to Coalter (39, 40), many sports-based interventions merely possess a vague representation of how a particular impact can be established. Scholars have also argued that research on the developmental potential of sport is predominantly focused on individual outcomes instead of how and why any development occurs (55). This has led researchers such as Harris (81) to refer to SfD as “a field defined by its claims as opposed to its results” (p. 796). Instead, the exercise of explicating the underlying assumptions forces program designers and practitioners to think more profoundly about the intervention (50). It thereby offers the possibility to question the assumed logic and arrive at a more realistic and coherent intervention.

Third, and not entirely unexpected, the imprecise formulation of desired outcomes had implications for the M&E of participants' progress. Despite several promising elements regarding tracking participants' progress, there was no coherent strategy to address the different stages (needs analysis, goal setting and M&E of progress) that participants go through during the program. This resonates with findings from a European study on the contribution of sport to the employability of NEETs (82), which concluded that SfE programs devote only minimal attention to the follow-up or measurement of participants' progress.

### Determining sources of the lack of a systematic approach

A number of possible sources for the lack of a systematic approach can be suggested. First, program providers may underestimate the importance of defining precisely both specific outcomes and accompanying strategies to intentionally facilitate those outcomes. Respondents organizing the female program in our study reported that the role of sport was not a subject of reflection when designing the program. One of the partners, responsible for teaching the sport sessions, was unaware of why sport was part of the program. It suggests that the main program organizers had only minimal reflection on what they are doing with sport, how they are doing it and why. In addition, they did not inform the partner responsible for teaching these sessions. This lack of consideration might be related to a general belief in the power of sport in which sport will unconditionally benefit the employability level of the participants. Sport is often considered as “a unitary experience” (83) (p. 136) whereby its positive outcomes are taken for granted (49). This essentialist view on sport neglects existing knowledge which indicates that its benefits are dependent on how sport is implemented and how it is experienced by its participants (83). Not the program or sport generates the aimed outcome of employability, it is mechanisms or underlying theories which are responsible for the success of a program (56).

Second, even if program developers are aware of the relevance of clearly defining outcomes and program processes, they might lack specific knowledge and frameworks to successfully complete this task of outlining and implementing. According to Weiss (56), constructing a program theory can be considered as an analytical task that differs from the “empathetic, responsive, and intuitive stance of many practitioners” (p. 87). Moreover, it requires practitioners to question their own initiative and find a consensus with fellow program providers about what they are trying to achieve and how. Blades et al. (13) also pointed out that there is variation with regard to defining and measuring employability. This ambiguous nature of employability, in addition to a possibly limited understanding of M&E, may also account for the lack of a systematic approach with regard to the follow-up of participants' progress and eventually assessing whether someone is ready to enter the employment market. Moreover, findings suggest additional knowledge is needed to optimally apply sport as a tool for experiential learning. Coaches offering the sport plus sessions within the male program reported that offering intentionally designed sporting experiences to assist in the development of skills was difficult. The male program organized one sport session where participants had to lead a boxing session and where an explicit link was made with job interview skills. But our findings revealed that specific knowledge was missing to apply such an approach systematically on all other sport sessions.

Third, although the program in our study consisted of a cooperation between several partners, the adopted program strategy was not discussed and aligned among these partners. The failure to do so prevented working from shared program aims, supported by all partners. Several examples in the present study show evidence of a lack of integration of the various program activities. A notable illustration relates to the different guidance styles used by different program providers. The male program was characterized by different approaches regarding the method and content of the program (experiential learning vs. direct instruction). These different approaches had a hampering effect on the general cohesion of the project as coaches withdrew themselves from activities they did not support. In parallel, the interviewees reported that the imbalance in critical support between the different coaches (critical support vs. unconditional support) caused the program to fall short in quality and is therefore a proper example of what Weiss (50) would denote as “working at cross-purposes” (p. 517) due to different ideas about how the program is supposed to work. The fact that they perceive these differences as conflicting instead of complementary, points to the lack of an overarching vision. This common understanding is, however, essential in social programming (50), especially when different partners are involved (84). Therefore, the creation of a theory of change might address this issue as its construction presupposes that involved stakeholders (e.g., managers, practitioners, funders, employers) discuss different perspectives and attempt to reach consensus (49, 56). This might avoid a scenario whereby different practitioners contradict each other (e.g., the different and allegedly conflicting guidance styles within the present case study) but instead, increases the focus of the intervention.

A final determining factor for the lack of a systematic approach concerns the pressure to comply with the requirements of funders. As providers reported pressure from local authorities to provide simple and quantitative evidence and to put a focus on positive results, the present study confirms the pressure of sports-based employability programs to deliver funder-favored numerical outcomes (34). Moreover, this pressure was accompanied by a tendency among program providers to formulate ambitious objectives (i.e., gaining paid employment). This is, according to Coalter (49), a more general phenomenon among funder-dependent SfD organizations. As a result, a divergence appeared to exist between the ambitious program objectives and the actual delivery of the program. To illustrate, attaining the presumed objective of employment is rather unlikely as the program focuses solely on participants' own ability and does not take into account broader structural dimensions such as housing and the local labor market (34). Therefore, in order to deliver a coherent program, it would be vital to either (a) adjust the content of the program in line with the objectives expected by funders (if feasible) or (b) attempt to initiate a discussion with funders whereby one tries to manage their expectations. Weiss (50) argues that such a conversation is facilitated by a theory of change as it enables the acknowledgment of unrealistic assumptions. Moreover, she states that local policy makers, responsible for program funding, are interested in explanatory stories such as those that can be provided by a theory of change.

### Advancing the use of theory-based approaches

Based on this study, several applied and research implications can be derived. First, as stated, it is advantageous to adopt a theory-based approach when implementing or conducting research into SfE programs. Practically, and in line with a theory of change approach as outlined by Weiss (56), this implies that SfE programs should attempt to precisely outline their desired outcomes and impacts in line with the problem they want to tackle and articulate what necessary outcomes are required to achieve this impact (57). In turn, these outcomes should be connected with the program's outputs, activities or interventions and resources. According to Weiss (50, 56), even the act of explicating assumptions can be considered as developmental as this exercise offers the possibility to identify gaps in the intervention whereby the chain of reasoning cannot be logically expected based on existing knowledge and experience. To start with, organizations can use the recently developed tentative generalizable “program theory” or “theory of change” for SfE programs of Coalter et al. (62). The theory outlines different program elements that have the potential to maximize successful program implementation and delivery. The authors suggest that organizations can use this program theory as “a template to enable providers to think about how they recruit, design and deliver their programs and seek to define and achieve their outcomes” (62) (p. 17).

Second, future work should advance our understanding of generative mechanisms that operate within SfE programs. Generative mechanisms entail the mechanisms that underpin the relationship between complex social interventions and their outcomes (85). Within each social intervention, different outcomes are produced for certain people, in certain circumstances. Upon evidence of some of these context, mechanism and outcome configurations or theories, refined theories can be obtained by testing them within other social programs (57). As such, it is referred to as ‘generative’ mechanisms as these mechanisms explain why an individual or a group of individuals react in a particular way. We thereby concur with the position of Pawson (75) and Coalter (39), who call for a fundamental shift from research that needs to prove sports' added value to understanding “the social processes and mechanisms that might lead to desired outcomes for some participants or some organizations in certain circumstances” (40) (p. 311). As such, the same mechanisms that might lead to effective program delivery can be present and triggered in what at first sight appear to be different programs (49, 50, 78). More detailed insight into these mechanisms might therefore enable future programs to build on this knowledge when designing activities and constructing their theory of change. Although the proposed program theory of Coalter et al. (62) provides a step forward, the authors invite others to “interrogate, disagree, and/or develop the proposed program theory through practice and research” (p. 17).

Third, when adopting a theory-based approach, it becomes possible for organizations and researchers to test the specified assumptions and measure desired outcomes directly related to the program (50, 52). To this end, future studies can monitor and evaluate the delivery and effectiveness of SfE programs, in order to help organizations to offer a more systematic program. In line with a conceptualization of employability as a term with slightly changing definitions and measurements (13), this study confirmed that the follow-up or measurement of participants' progress can be considered as a complex endeavor. Therefore, more insight into employability measures might enable SfE organizations to better define outcomes and impact, measure the impact of the program, make an initial objective assessment of participants' qualities and weaknesses, and provide a basis for mentoring discussions. Recently, Coalter et al. (86) contributed to this need by developing a monitoring and evaluation manual for SfE programs. The manual is based on the idea that proper M&E of programs can only take place when one first gets an understanding on how the program is expected to contribute to its outcomes and impacts.

Fourth, in line with the need for a better understanding of how SfE programs work, future work should acquire more specific knowledge and expertise on how SfE programs can organize ‘sport plus’ sessions. As indicated, using sport as a tool to teach soft skills requires programs to go beyond traditional sport sessions and draw on the approach of experiential learning (39). However, our analysis suggests that program providers may not have sufficient expertise to design and coach such targeted sport sessions. This is not entirely unexpected, as according to Theeboom et al. (87) sport coaches and physical education teachers are trained to teach specific sporting skills including technical and tactical knowledge, rather than providing life skills development trough sport. To address this, future research might look at relevant principles within, for example, youth work and at how these principles can be implemented by SfE program providers.

### Limitations

This study has a number of limitations that should be acknowledged. First, the presented findings are based on program providers' perspectives only. Although our aim was to investigate how organizers construct and deliver their program, obtaining information from other stakeholders such as participants and funders might have increased our understanding of how the program was constructed and delivered. Second, as this study was exploratory in nature, we opted for a rich description of a phenomenon (70). This also implies that statistical-probabilistic generalizability to other SfE programs may be difficult. Instead, the thick description of a purposefully selected case should enable a possible transfer to other contexts. Third, it is important to note that reflecting on the strategy of using sport to facilitate employability presumes that sustainable jobs are available. This is regularly referred to as an challenge in several localities (8, 34).

## Conclusion

To summarize, the present study was an attempt to gain insight into a particular and already existing SfE program. We specifically explored how it constructed and delivered its program. This question was prompted by the limited amount of research within the area, despite the large and growing interest in using sport to increase employability among NEETs (36, 88). Our case study analysis showed that, consistent with more general SfD programs, a SfE program might struggle with offering a systematic approach. Several possible reasons that could produce these challenges were discussed. In order not to organize SfE programs merely on the basis of intuition and experience and arrive at a more structured and systematic program, we discussed, in general terms, the use of a more theoretically informed and systematic approach.

## Data availability statement

The datasets presented in this article are not readily available because it concerns confidential data. We consciously opted to keep the selected case and its participants anonymous within this study. Requests to access the datasets should be directed to TC, tessa.commers@vub.be.

## Ethics statement

The studies involving human participants were reviewed and approved by Ethical Committee for Human Sciences - Vrije Universiteit Brussel. The patients/participants provided their written informed consent to participate in this study.

## Author contributions

TC, MT, and FC jointly conceptualized the study. The data collection and data analysis was conducted primarily by TC, with MT acting as a critical friend and supervisor who provided regular feedback. TC wrote the first draft of the manuscript, with MT and FC providing extensive feedback. All authors contributed to the article and approved the submitted version.

## Funding

Funding was provided through external financing by a Flemish municipality.

## Conflict of interest

The authors declare that the research was conducted in the absence of any commercial or financial relationships that could be construed as a potential conflict of interest.

## Publisher's note

All claims expressed in this article are solely those of the authors and do not necessarily represent those of their affiliated organizations, or those of the publisher, the editors and the reviewers. Any product that may be evaluated in this article, or claim that may be made by its manufacturer, is not guaranteed or endorsed by the publisher.
